# CBCT evaluation of root canal morphology and anatomical relationship of root of maxillary second premolar to maxillary sinus in a western Chinese population

**DOI:** 10.1186/s12903-021-01714-w

**Published:** 2021-07-20

**Authors:** Yujia Yan, JingLin Li, Hualing Zhu, Jun Liu, Jiayin Ren, Ling Zou

**Affiliations:** 1 State Key Laboratory of Oral Diseases, National Clinical Research Center for Oral Diseases, Department of Endodontics, West China Hospital of Stomatology, Sichuan University, Chengdu, 610041 China; 2 State Key Laboratory of Oral Diseases, National Clinical Research Center for Oral Diseases, Department of Orthodontics, West China Hospital of Stomatology, Sichuan University, Chengdu, 610041 China; 3 State Key Laboratory of Oral Diseases, National Clinical Research Center for Oral Diseases, Department of Oral Radiology, West China Hospital of Stomatology, Sichuan University, Chengdu, 610041 China

**Keywords:** The second permanent maxillary premolar, Root canal system, Cone-beam computed tomographic (CBCT), Anatomical relationship, Maxillary sinus

## Abstract

**Background:**

To evaluate the root anatomy, root canal morphology and the anatomical relationship between the roots and maxillary sinus of maxillary second premolars by CBCT in a western Chinese population.

**Methods:**

A total of 1118 CBCT scans of the maxillary second premolars were collected from West China Hospital of Stomatology, Sichuan University. Information below were measured on axial, coronal and sagittal sections, recorded and evaluated properly: the number of roots and canals, the morphology of canal system classified by Vertucci standard, the inter-orifice distance of canal orifices, the curvature of each canal and the distance from root tip to maxillary sinus floor.

**Results:**

Among the 1118 teeth, 94.2% (1053) are single-rooted and 55.1% (616) have one canal. Type I (55.1%) is the commonest root canal morphology followed by Type II (31.9%). The mean inter-orifice distance (IOD) for multi-canal teeth ranging from 2.72 ± 0.32 to 3.41 ± 0.11 mm. Of 1622 canals, 38.8% (630) curvature are mesiodistal and 30.9% (501) are straight canals. The distance from root tip to maxillary sinus floor increased with age and the mean distance of single-rooted ones is 2.47 ± 3.45 mm.

**Conclusions:**

All kinds of canal morphology category can be detected in maxillary second premolars. The IOD might be a predictable factor for root canal morphology. Roots of maxillary second premolars are related to maxillary sinus which should be treated carefully.

## Background

Maxillary second premolars seem to be insignificant in dental arch, not as important as the first molars which guide the occlusal foundation, or contributing a lot into our smile like the anterior teeth. Nevertheless, studies showed that the maxillary second premolars may suffer different kinds of diseases like granuloma [1] or dens evaginatus [2, 3], for which some even claimed that the maxillary second premolars are one of the most frequently endodontically treated maxillary teeth [4]. Furthermore, the premolars happen to be the favor choice for extraction cases for orthodontic treatment [5, 6], which confronts the clinicians with dilemmas since the root could be close to or even wrapped by the maxillary sinus floor.

When various pathological factors mentioned above triggered the dental pulp inflammation, root canal treatment would be the first adopted routine therapy. Efficient root canal preparation is crucial for a successful endodontics treatment, which requires clinicians’ adequate comprehension of root canal morphology. Most periapical X-ray films might show the maxillary second premolars with one root and one canal. However, maxillary premolars have a highly variable internal canal configuration, which can vary according to race and geographic origin [7]. Amounts of researches have reported maxillary second premolars with more than one root or one canal [8, 9]. The incidence of three canals in maxillary premolars has also been reported to vary from 0 to 10% [10]. Therefore, evalution of root morphology and root canal mophology ahead of treatment is sort of important for therapy.

Though the implications of root form and root canal morphology on clinical endodontic have been fully established in western literature, the features of root canal morphology in Asian settings have not been well documented. Among Malaysian subpopulation, Pakistani population and Saudi population, studies show that maxillary second premolars mainly have one root (84.3–91.9%) and the commonest canal morphology is Vertucci Type I (49.4–58.2%) [11–13]. Meanwhile, published researches on different Chinese subpopulation drew the similar conclusions. A great fluctuate of detection rate cannot be ignored which might get influenced by the difference of subpopulation. Additionally, those subpopulation observed mostly are distributed in coastal eastern and southern China [14–17]. We all know that western China is a multiple ethnic region, therefore, it is of great clinical value to study the root canal morphology of maxillary second premolars in this region representative of China.

The maxillary sinus belongs to the paranasal sinuses which stop growth at approximately 20 years old. With the sinus floor extending to adjacent roots, the roots could irrupt into the sinus and get wrapped. Anatomically close relationship results in a functional connection between maxillary sinus and roots. An inappropriate anatomical relationship might block the movement of orthodontic tooth. Meanwhile, debris pushed out by excessive flush during root canal treatment or microbial infections from tooth carious cavity all can possibly cause an odontogenic maxillary sinusitis. Roots of maxillary second premolars are close to the maxillary sinus. Therefore, cognition of the anatomical relationship between the maxillary sinus floor and the root of maxillary second premolars is vital to avoid odontogenic damages in whether tooth extraction, orthodontic or endodontic treatment [18–21].

Oral cone-beam computed tomography (CBCT) examination is a quick, convenient and noninvasive method which can help dentists quickly learn about the anatomical profiles of target tooth while reducing the health and financial cost into the lowest. Besides that, the CBCT scans could reconstruct the three-dimensional simulation, which reflects the circumstance of root and adjacent anatomical structures. This study also aims to value the distance from root tip from maxillary second premolar to the maxillary sinus floor with the assistance of CBCT [22–25].

## Methods

### Patients

Sample calculation was based on single sample rate calculation formula: n = ${\text{n}} = \left( {\frac{{Z_{\alpha } }}{\delta }} \right)^{2}\uppi \left( {1 -\uppi } \right)$ [26]. The overall Vertucci type I prevalence π = 50.3%, α = 0.05, δ = 0.05, one-tailed, where π is from previous studies [16] using 95% confidence intervals. As for the anatomical relationship with maxillary sinus, the overall prevalence of root contacting the maxillary sinus floor, π = 20.2%, α = 0.05, δ = 0.05, one-tailed, where π is from previous studies [20] using 95% confidence intervals. To get a higher precision, we have enlarged the larger result calculated by the above formula by 10% as the final minimized sample size, which is 296. All cone-beam computed tomographic(CBCT) data were collected at the department of radiology of West China Hospital of Stomatology, Sichuan University from January 2017 to February 2020, among patients who have endodontic treatment needs (pulptitis, pulp exposure, apical periodontitis, dental trauma, etc.). We have also sifted through all the candidates’ information to separate those who came to the department of endodontics for the first time as samples, which consists of 709 patients, ending up with 559 patients (347 males and 212 females) while excluding 150 patients. Based on that, 1118 bilateral maxillary second premolars were screened out with each patient’s basic information recorded, such as name, gender and age. The study was approved by the Medical Ethics Committee of West China Stomatology Hospital of Sichuan University with the approval number: WCHSIRB-D-2020-437.

Whole data have been screened according to criteria below to avoid misleading by image artifacts, man-made changes or teeth moving.

1. No dental trauma or dysplasia (fusion, central cusp deformity, dens invaginatus, etc.)
2. No periapical lesions or orthodontic treatment
3. No previous root canal treatment or post- or crown restoration
4. Mature root apical foramen without root resorption or calcification
5. No missing adjacent or opposite jaw tooth
6. No maxillary deformity, trauma or maxillofacial tumor
7. Qualified CBCT scans

### Radiographic techniques

All CBCT scans were scanned using a CBCT device (3-dimension Accuitomo, J.MORITA MFG. CORP. Kyoto Japan), with those exposure parameters: 85 Kvp, 4.0 mA, 17.5 s scan time, with voxel size of 0.25 mm, scanning angles of 360° and field of view of 60 mm * 60 mm for all images. Those images were shoot by an experienced technician following the manufacturer’s instructions with lowest dose radiation.

### Calibration

Calibration for the study was performed between the observer and an expert oral radiologist. The observer was trained and calibrated for reading the CBCT images in a pilot study with a sample size of 50. The observer evaluated the CBCT images using axial, sagittal and coronal views to identify root and root canal morphology and anatomical relationship of root of maxillary second premolar to maxillary sinus. Disagreements were discussed, and a consensus was reached after discussion. After the calibration, test for inter and intra examiner errors were performed. The kappa value is 0.824 (*p* = 0.000000 < 0.05) and the ICC is 0.957 (*p* = 0.000000 < 0.05). Two-way ANOVA was used for the intra examiner errors since each measurement was performed three times. Different patient were different blocks and the three measurements were the different groups. The *p*-value of variation of groups for each examiner is 0.940 and 0.721 respectively.

### Evaluation of the images

The RadiAnt DICOM Viewer software (64 bit, 2020.2.3) was used as the image reconstructing and measuring tool. Two endodontists have measured all the images individually with an oral radiologist’s opinion as the final golden standard when inevitable disagree on the same images. Views of maxillary second premolars from pulp chamber to apical foramen on the coronal, sagittal and axial sections were observed to analyze the root canal morphology. Data of teeth on both sides were measured and recorded: the root number and morphology, the number and configuration of canal, the inter-canal distance of canal orifices, the distance from each root to the maxillary sinus floor and the curvature of each canal in both buccopalatal and mesiodistal direction. The methods of Zhang [27] was used to classify the curvature degree of each canal, while the distance was divided by the method of Shahbazian [28].

SPSS 21.0 software (SPSS, Inc., Chicago, IL, USA) was used for statistical analysis. Descriptive statistics was used to describe the number of roots and root canals, as well as the detection rate of different root canal morphology. Independent samples t test was used for inter-orifice distance and curvature on both mesiodistal and buccopalatal sides. ANOVA test was used for the distance from root tip to maxillary sinus floor for different groups of age and different roots. Chi-square test was used for the detection rate of different classification on the distance from root tip to maxillary sinus floor in both genders. Statistically significant differences were defined at *p* < 0.05.

## Results

### The morphology of root and canal of maxillary second premolars

In this study, only 65 (5.8%) teeth have two roots, while most of the teeth were one rooted (94.2%). In terms of root canals, single canal (55.1%) is higher than two canals (44.7%) and three canals (0.2%). Type I canal configuration (1–1, 55.1%) is the most prevalent in maxillary second premolars, followed by Type II (2–1, 31.9%). Root canal category is highly conserved in one canal teeth (Type I, 100%) and three canals teeth (Type VIII, 100%). Meanwhile, other root canal categories except Type I and VIII were all detected in double canals teeth while Type II (71.4%) is the commonest followed by Type IV (22.8%). The detection rates of different canal category varies with gender and root number were listed in Tables 1 and 2. As for those multi-rooted teeth, the root furcation often located near the apical region (53.8%).

**Table 1:** The number of roots and different canal category in the maxillary second premolars [n, (%)]

Root number	Canal category							
	Type I	Type II	Type III	Type IV	Type V	Type VI	Type VII	Type VIII
One root	616 (55.1)	357 (31.9)	6 (0.6)	52 (4.7)	16 (1.4)	5 (0.4)	1 (0.1)	0
Two roots	0	0	0	62 (5.5)	1 (0.1)	0	0	2 (0.2)
Total	616 (55.1)	357 (31.9)	6 (0.6)	114 (10.2)	17 (1.5)	5 (0.4)	1 (0.1)	2 (0.2)

**Table 2:** Detection rates of different canal category in both gender [n, (%)]

Gender	Canal category							
	Type I	Type II	Type III	Type IV	Type V	Type VI	Type VII	Type VIII
Female	232 (54.7)	141 (33.3)	3 (0.7)	44 (10.4)	4 (0.9)	0	0	0
Male	384 (55.3)	216 (31.1)	3 (0.4)	70 (10.1)	13 (1.9)	5 (0.7)	1 (0.2)	2 (0.3)

Since almost half of the maxillary second premolars were observed with more than one canal orifice on the pulp chamber floor, the distance between those canal orifices also got measured. The amount of type VI and type VIII is too small to cause bias within statistical analysis, thus only the average distances of type II and type IV are compared. Results are listed in Table 3.

**Table 3:** The distance between canal orifices

nal morphology	$\overline{X} \pm S$(mm)
Type II	2.76 ± 0.55
Type IV	3.02 ± 0.55^a^
Type VI	2.72 ± 0.32
Type VIII	3.41 ± 0.11

*p < 0.05, independent samples *t* test, type VI and type VIII not included

^a^Significant difference, *p* = 0.000011 < 0.05

### The curvature of the maxillary second premolar canals

According to the method of Zhang, the canal with curvature less than 10° is defined as straight canal, while those with more than one curve are defined as S-shaped root canals. The commonest curvature orientation of maxillary second premolars is mesiodistal (38.8%). Meanwhile, the straight one (30.9%) is almost as common as the buccopalatal (27.6%), both much more frequently detected than S-shaped ones (2.7%). However, the average curvature and ratio of severely curved canal(> 25°) on buccopalatal orientation is higher than mesiodistal ones. Table 4 shows more details.

**Table 4:** The curvature of root canal on its dominantly curved side and gring. *p < 0.05, independent samples *t* test

	Average curvature $\left[ {\overline{X} \pm S\left(^\circ \right)} \right]$	Curvature degree [n, (%)]	
Dominantly curved direction		Moderately curved (10–25°)	Severely curved (> 25°)
Mesiodistal	18.06 ± 10.39	494 (78.4)	136 (21.6)
Buccopalatal	21.36 ± 8.62^a^	321 (71.7)	127 (28.3)

^a^Significant difference, *p* = 0.000000 < 0.05

Besides, majority of all the curved teeth observed in this study exhibited a preference curving position in apical region (75.9%).

### The anatomical relationship between the root and maxillary sinus floor

**Fig. 1:**
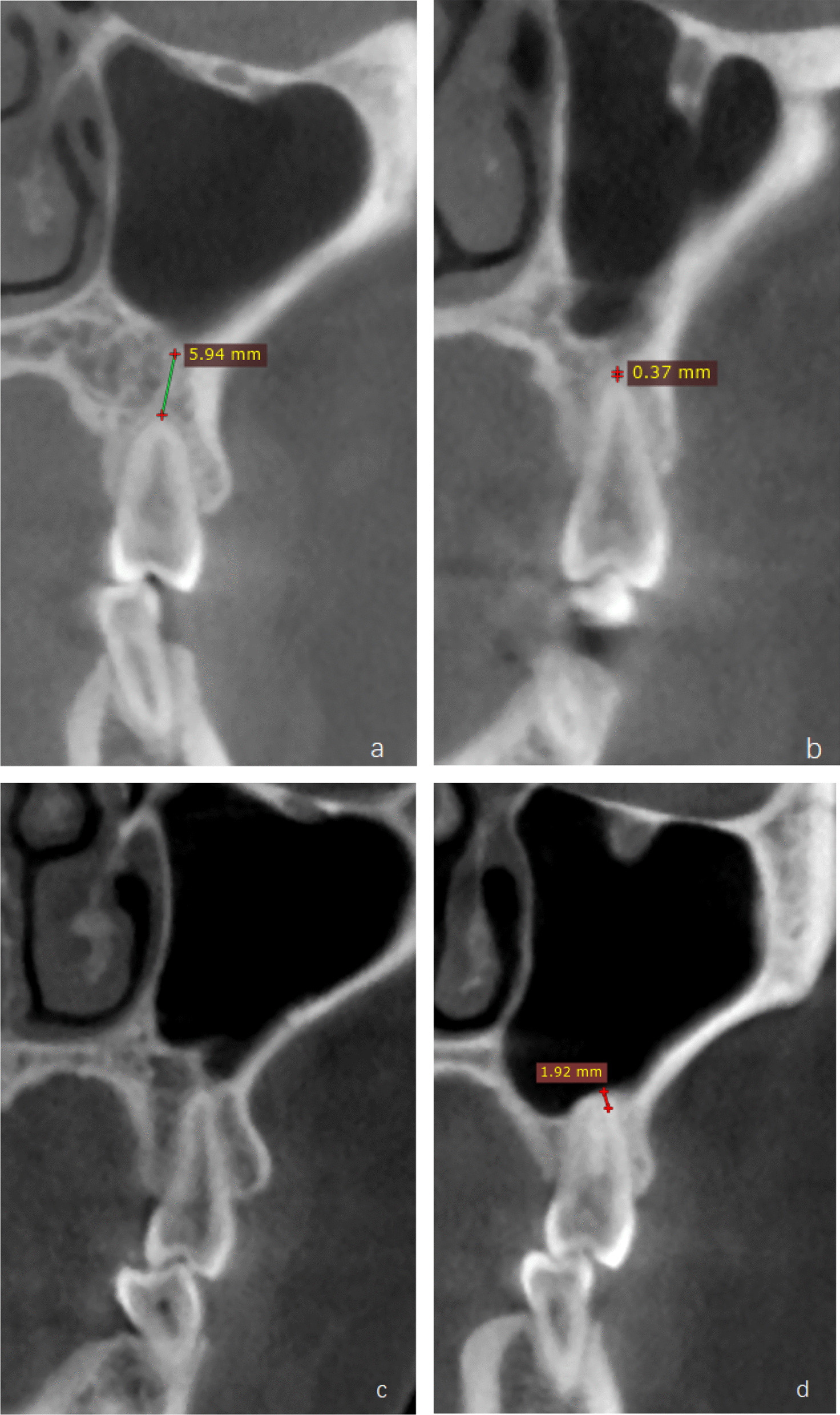
Distances from root tip to the maxillary sinus floor measured in the coronal section are divided into four types by the method of Shahbazian. **a** Distance larger than 0.5 mm; **b** distance less than or equal to 0.5 mm but larger than 0 mm; **c** distance equal to 0 mm, root tip attached to the sinus floor; **d** roots protruded into the sinus, distance recorded as negative

This study devided the distance between the root and maxillary sinus floor into four types according to the method of Shahbazian [28]. Detection rates of each type of both genders are presented in Table 5. Considering the maxillary sinus might narrow with age, comparison between different age groups has been taken to find whether the age might influence the anatomical relationship, of which the results are showed in Table 6 (Fig. 1; Table 7).

**Table 5:** Types of the distance form root to the maxillary sinus floor for both gender

	Type of distance from root to maxillary sinus floor [n,(%)]			
Gender	Type I > (0.5 mm)	Type II (≤ 0.05 mm)	Type III (= 0 mm)	Type IV (< 0 mm)
Female	268 (22.7)	25 (2.1)	133 (11.2)	19 (1.6)
Male	424 (35.8)	50 (4.2)	161 (13.7)	103 (8.7)
Total	692 (58.5)	75 (6.3)	294 (24.9)^a^	122 (10.3)^b^

*p < 0.05, chi-square test

^a^Significant difference, *p* = 0.002 < 0.05

^b^Significant difference, *p* = 0.000000 < 0.05

**Table 6:** Distances from root to the maxillary sinus floor in different age groups

	$$\overline{X} \pm S$$(mm)
Age group (y)	
16–23	0.79 ± 2.42^a^
24–35	1.94 ± 2.88^b^
36–45	3.28 ± 3.59^c^
46–55	3.65 ± 4.07^c^
56–	4.21 ± 3.57^c^

Different letters symbolize the statistic difference, *p* < 0.05, ANOVA test

Different superscript letter means different subsets which has magnificent statistic difference. The *p*-value between a and b is 0.000001 < 0.05; the *p*-value between a and c is 0.000000 < 0.05; the *p*-value between b and c is 0.000010 < 0.05

**Table 7:** Distances from root to the maxillary sinus floor. Different letters symbolize the statistic difference, *p* < 0.05, ANOVA test

	$$\overline{X }\pm S$$(mm)
Second premolar: single root	2.47 ± 3.45^a^
Second premolar: buccal root	1.49 ± 3.21^b^
Second premolar: palatal root	2.13 ± 3.13^ab^

Different superscript letter means different subsets which has magnificent statistic difference. The *p*-value between a and b is 0.025 < 0.05

## Discussion

In this study, an overwhelming majority of maxillary second premolars were observed with one root (94.2%).This incidence seemingly kind of shifted with race since some previous researches have reported a considerably lower rate, such as in Jordanian population (55.3%) [29], South African subpopulation (78.2%) [30], Spanish population (82.9%) [7], Saudi population (85.2%) [12], Iranian population (91%) [31] and Turkish Cypriot population (91.9%) [32]. It’s worth noticing that researches done before by Hu (95.2%) [17] and Li (96.2%) [16] in Chinese subpopulation also got the similar result. Conclusion that single-rooted second premolars are a Mongoloid trait by Neelakantan might count on this situation [33].

The most frequently detected canal morphology was Type I (55.1%), whereas Type II (31.9%) was the commonest in multi-canal category. Interestingly, double-rooted premolars showed a high conservatism on canal morphology, 95.4% for Type IV, while single-rooted premolars have abundant canal categories, 58.5% for Type I and 33.9% for Type II. The ratio of single-canal and double-canal is debatable likewise. Researches in foreign ethnics mentioned above showed a detection rate of one canal less than 50%, which reasonable based on the low single-rooted ratio. However, double-canal type was the superior category in some studies ranging from 54.3 to 85.7% [14, 29] unlike Li’s study (48.9%) [16] and the present study (44.7%). All classification of Vertucci canal morphology were found in this study. No significant statistical difference was observed between genders. Additionally, two double-rooted premolars were found with three canals, one in palatal root and two in buccal root. This condition is rare because three canals usually detected in three-rooted teeth in plenty of previous studies [29, 34]. Cases like that might lead to a missing canal.

The inter-orifice distances (IOD) are measured at the level of pulp chamber floor. IOD of Type IV (3.02 ± 0.55 mm) was larger than IOD of Type II (2.76 ± 0.55 mm) and there was statistical difference. A study on mandibular first molars also investigated the connection between IOD and canal morphology, which reported with IOD larger than 3 mm the canal was more likely to be Type IV [35]. Wei [15] also found the ratio in Type IV of IOD larger than 3 mm was higher than in Type II in the study of maxillary premolars of Chinese population. The IOD increasing might cause the fault of two canals fusion. Clinicians may conjecture the canal morphology opening the pulp chamber before get a fluent pathway.

Curvature of canals was measured by the method of Zhang [27]. Compared to the method of Schineider [36], the marks for measuring are the same but a looser curvature classification standard is more suitable for larger curved angle. Maxillary second premolars usually present a mesiodistal curve (38.8%) with a moderate mean curvature (18.06° ± 10.39°), whereas the less frequently buccopalatal curved ones have a severe mean curvature (21.36° ± 8.62°). The curvature tendency was similar to Wei’s study [15], mesiodistal superior to buccopalatal, since the maxillary sinus floor might exert a mesially pushing force on the root. However, the larger average curvature of buccopalatal orientation indicated that some roots might have a large curvature on the buccopalatal orientation. In Jang’s research [37], the distance of maxillary second premolars from root apices to palatal cortical walls is twice bigger than those to buccal cortical walls. This magnificent distance difference might cause an imbalance of power during the development of bone and root, which results in a large curvature on the buccopalatal orientation.

**Fig. 2:**
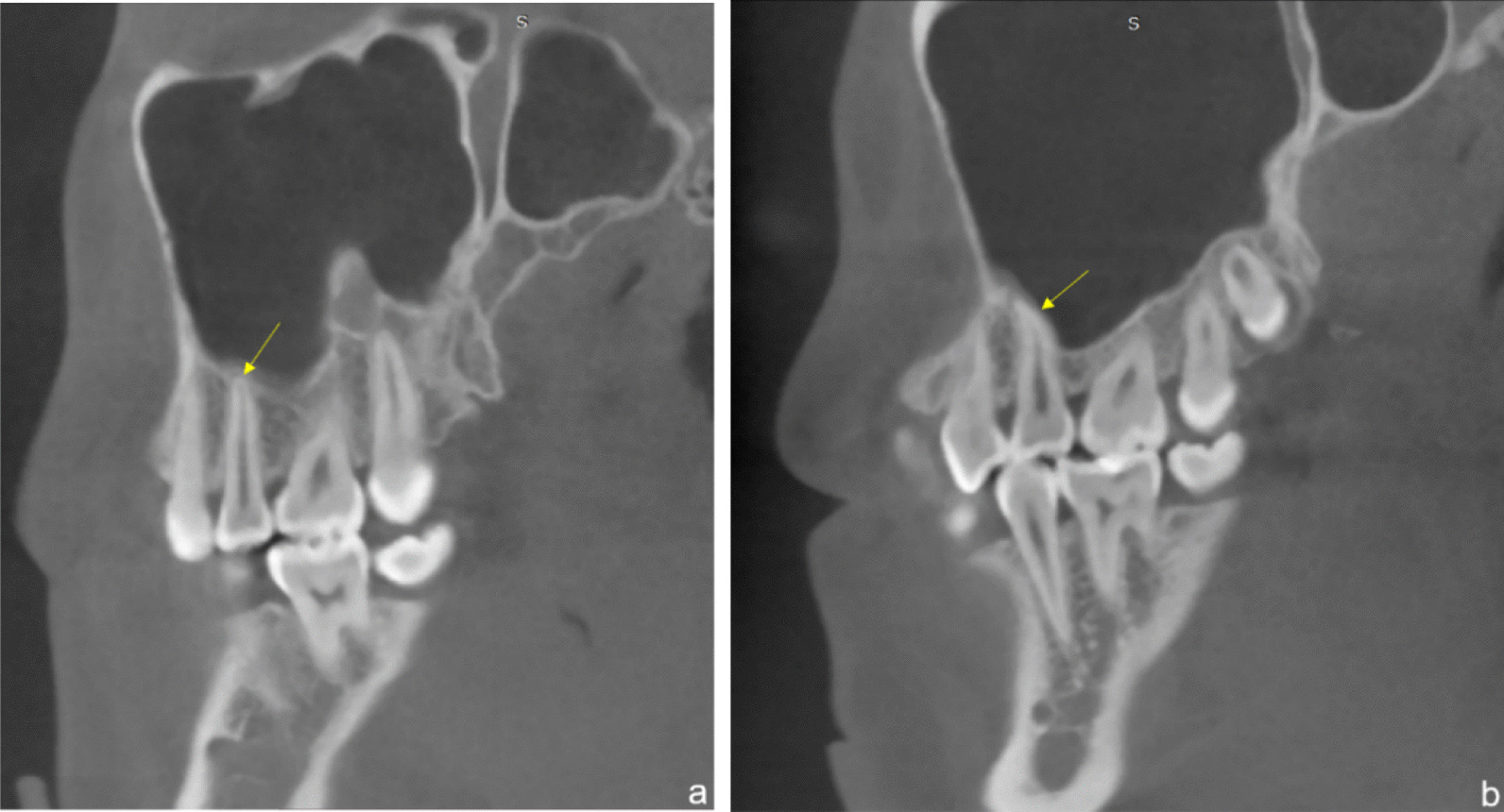
Type III according to the classification of Shahbazian. When the roots exactly adjoined the maxillary sinus floor, the curvature of root in the sagittal section. **a** The root is straight, **b** the root is mesially curved (17.7% of all type III cases)

Distance from root tip to maxillary sinus floor were measured by the method of Shahbazian [28]. The distance from the buccal and palatal root tip of the two-rooted maxillary second premolar to the maxillary sinus floor were 1.49 ± 3.21 mm and 2.13 ± 3.13 mm respectively, which indicated the buccal root is much closer to the maxillary sinus floor than the palatal one. As for the single-rooted maxillary second premolars, they seem to be approximately close (2.47 ± 3.45 mm) to the maxillary sinus floor compared to the palatal root of two-rooted second premolars. Some studies also measured the distance in group of different roots [15, 37–40]. The distance ranges from 1.99 ± 1.84 to 5.29 ± 2.47 mm with the same tendency, palatal root further than buccal one. That makes sense if the ratio of different age group in those studies is different since the maxillary sinus floor might move upward out of aging, which increases the distance. Previous study found that maxillary sinus grows by pneumatization until the eruption of the third molars at approximately 20 years old [40–43]. After that growth period, the volume of maxillary sinus may be reduced with the maxillary sinus floor going upward unless some interference is encountered [44–47]. Meanwhile, our colleagues in orthodontics bring up a hypothesis based on their clinic experience that when the roots of maxillary second premolars exactly adjoined the maxillary sinus floor, the roots seem to quite likely get pressed so that they were pushed to the mesial side (Fig. 2). To verify that hypothesis, we go through all the Type III images and found that the mesially-pushed detection rate is 17.7%.

In order to verify whether age is an influence factor on the distance, all patients were divided into five groups by age. Results indicated that distance actually increased by age indeed. After transforming the distance into classification, we found that 64.8% maxillary second premolars (Type I and Type II) were not attached to the maxillary sinus, overwhelming the rate of Type III (24.9%) and Type IV (10.3%). In spite of different kinds of classification standard, results of researches all turned out to be the fact that most of the roots of maxillary second premolars are safely away from maxillary sinus floor [20, 39, 40].

There are some limitations in our study. Firstly, sample size, especially the number of different age isn't approximately equal as designed ideally, which might enlarge the difference between groups. Secondly, there may be some other types of canal configuration which don't fit in the eight variations of Vertucci's Classification. For this reason, the classification proposed by Ahmed et al. could be a better choice in our further research [48]. Finally, in consideration of the complex ethnic composition in western China, further study could divided the patients into groups by ethnic strictly.

## Conclusions

In conclusion, the commonest morphology type of maxillary second premolar in western Chinese subpopulation was single rooted with one canal while all the other root canal morphology types could be found. Besides that, most root canals were mesiodistally curved to a moderate degree. The distance from root tip to maxillary sinus floor increased with age.

## Data Availability

All the datasets used and analyzed during the current study are available from the corresponding author on reasonable request.

## Abbreviations

IOD: Inter-orifice distances
CBCT: Cone-beam computed tomography
BP: Buccopalatal
MD: Mesiodistal
